# Determining the levels of pelvic floor disorders in women diagnosed with urinary incontinence: a mix-method study

**DOI:** 10.3389/fmed.2024.1509973

**Published:** 2025-01-30

**Authors:** Rüveyda Ölmez Yalazı, Nurdan Demirci

**Affiliations:** ^1^Marmara University, Institute of Health Sciences, Department of Obstetrics and Gynecology Nursing, Istanbul, Türkiye; ^2^Çanakkale Onsekiz Mart University, Faculty of Health Sciences, Division of Midwifery, Çanakkale, Türkiye; ^3^Marmara University, Faculty of Health Sciences, Division of Nursing, Department of Obstetrics and Gynecology Nursing, İstanbul, Türkiye

**Keywords:** urinary incontinence, pelvic floor dysfunction, mixed methods, psychosocial impact, quality of life

## Abstract

**Introduction:**

Urinary incontinence (UI) significantly affects women’s quality of life and may contribute to pelvic floor disorders. This study aimed to investigate the impact of UI on pelvic floor disorders through a mixed-methods approach.

**Methods:**

A convergent parallel design was employed, integrating quantitative and qualitative data. Quantitative data were collected using the “Global Pelvic Floor Disorders Questionnaire,” and semi-structured interviews were conducted for qualitative insights. Quantitative data were analyzed using SPSS 26.0, while qualitative data underwent content analysis with MAXQDA Pro 22 software.

**Results:**

Among the participants, 41.7% were diagnosed with stress urinary incontinence, which was associated with the highest level of discomfort. Qualitative findings revealed four main categories: effects on daily life, emotional and psychological effects, coping strategies, and treatment approaches and expectations. Participants reported that UI led to social isolation, reduced self-esteem, and increased anxiety.

**Discussion:**

The findings highlight that UI adversely affects both the physical and psychosocial well-being of women. Addressing this condition requires a holistic approach combining medical treatment with psychosocial support to mitigate its multifaceted impact.

## 1 Introduction

Urinary incontinence (UI), defined as the involuntary loss of urine, is classified into three types: stress, urge, and mixed UI, and is recognized as a significant social and health concern (1). The underlying causes of UI often involve dysfunction in the muscles and structures comprising the pelvic floor (2). UI affects 6–10% of women overall, approximately 10–25% of women over the age of 30, with prevalence increasing to 30–50% by the age of 50 (3, 4). Risk factors for UI include age, pregnancy, childbirth, and obesity (5–8). UI has a profound impact on women’s physical and mental health, disrupting daily activities and leading to morbidity and, in rare cases, mortality (9, 10). Consequently, early screening, assessment, intervention, and rehabilitation are essential components of managing UI effectively (11). Assessing the severity and discomfort caused by patients’ symptoms is critical for determining an appropriate treatment plan. This study aims to examine the effects of urinary incontinence on pelvic floor disorders and to evaluate the severity, frequency, and prevalence of these disorders using both quantitative and qualitative methods. By adopting this approach, the study seeks to gain a deeper understanding of the physical challenges faced by women diagnosed with UI and the extent of discomfort caused by these conditions.

## 2 Materials and methods

The study employed a convergent parallel design, a mixed-methods approach in which qualitative and quantitative data, given equal importance, are collected simultaneously and independently, analyzed separately, and integrated during the interpretation phase (12). The findings were reported in accordance with the Strengthening the Reporting of Observational Studies in Epidemiology (STROBE) guidelines (Supplementary File 1) and the Consolidated Criteria for Reporting Qualitative Research (COREQ) checklist (Supplementary File 2) (13, 14).

For the quantitative portion, a validated scale was used to assess the severity of pelvic floor symptoms in women with UI. Additionally, a questionnaire was employed to gather sociodemographic and obstetric information. For the qualitative portion, individual interviews were conducted via an online platform using Van Manen’s interpretive phenomenology model (15). The research was carried out between November 2023 and March 2024 after obtaining the necessary permissions.

In qualitative research, the sample size is determined by the principle of “saturation,” where data collection continues until concepts and themes begin to repeat and no new information emerges (12, 16). Accordingly, the qualitative portion of this study included 24 women. For the quantitative part, a power analysis conducted using G*Power 3.1.9.6 software, assuming 90% power and a Type I error of 0.05, determined the need for 240 participants.

Participants for both portions of the study were women aged 18–65, diagnosed with urinary incontinence, who had at least a primary school education, used the internet, and voluntarily agreed to participate. Quantitative data were collected using the “Personal Information Form” and the “Global Pelvic Floor Bother Questionnaire (GPFBQ),” while qualitative data were obtained through a semi-structured interview form designed specifically for women with UI.

The Personal Information Form was developed by the researchers based on the literature (17, 18). The GPFBQ, originally adapted into Turkish by Doğan and colleagues in 2016, measures symptoms and their severity related to stress, frequent and sudden urges to urinate, difficulty urinating, pelvic organ prolapse (POP), fecal incontinence, dyspareunia, and obstructive defecation (19). The GPFBQ includes nine items scored on a Likert scale, where women indicate symptom severity ranging from “not at all” (1) to “a great deal” (5). This practical and efficient tool is well-suited for both clinical and research settings. The Cronbach’s alpha value for the GPFBQ was 0.71 in the Turkish validity and reliability study and 0.82 in this study.

The semi-structured interview forms contained open-ended questions designed to guide the researchers during the interviews and capture participants’ views and experiences regarding pelvic floor health and related discomfort. A pilot study was conducted with five women diagnosed with UI to test the clarity of the data collection tools. Feedback from the pilot study informed adjustments to the interview forms. These participants were not included in the final sample.

The GPFBQ was digitized and distributed via an online survey platform. Researchers shared the online survey link with women meeting the study’s inclusion criteria, who were also invited to refer other women with UI and pelvic floor disorders fitting the criteria. Completing the forms took an average of 15 min.

The qualitative data were collected by researchers experienced and trained in qualitative methodologies. Informed consent was obtained prior to interviews, which were recorded and subsequently transcribed. Individual interviews were conducted face-to-face using the semi-structured interview form, with a moderator (ND) and a rapporteur (ROY) present. Each interview lasted approximately 30 min.

### 2.1 Statistical analyses

Qualitative and quantitative data were analyzed using distinct methods for each (12). Qualitative data were analyzed through content analysis. To ensure confidentiality, participants were coded with identifiers starting with “S” followed by their participant number. Researchers experienced in qualitative methodologies utilized MAXQDA Pro 22 software to code the data line by line and conduct content analysis. Themes and sub-themes were finalized during a collaborative meeting where researchers re-evaluated the data and reached a consensus.

Quantitative data were analyzed using SPSS 26.0. Descriptive statistics were employed to summarize the sociodemographic characteristics of the participants, and a *p*-value of <0.05 was considered statistically significant for all analyses. Logistic regression analysis was applied to account for the binary categorical structure of the dependent variable. Independent variables and other potential influencing factors were incorporated into the logistic regression model. The accuracy of the model was assessed using the Hosmer-Lemeshow test, with a significance level of *p* < 0.05 deemed acceptable. Odds ratio values and 95% confidence intervals were calculated to interpret the effect of independent variables on the dependent variable.

Additionally, Fleiss’s Kappa test was conducted to evaluate the agreement between responses on the GPFBQ scale. The chi-square test was also performed to assess relationships between categorical variables. Informed consent was obtained from participants prior to their inclusion in the study.

## 3 Results

The average age of the women who participated in the qualitative interviews was 45.3 ± 10.2 years; 27.7% had a university degree, 79.2% had a history of pregnancy, 37.5% were overweight, and 41.7% were diagnosed with stress urinary incontinence (Table 1).

**Table 1:** Descriptive characteristics of women (*n*: 240).

Variable	N	%
Age		
Mean ± SD	55.3 ± 10.2	
Educational level		
Primary education	80	33.3
Secondary education	70	29.2
Higher education	90	37.5
Pregnancy history		
Yes	190	79.2
No	50	20.8
Number of births		
None	50	20.8
1–2	90	37.5
3 or more	50	20.8
Body mass index (BMI)		
Underweight	30	12.5
Normal weight	80	33.3
Overweight	90	37.5
Obese	40	16.7
Type of urinary incontinence		
Stress UI	100	41.7
Urge UI	70	29.2
Mixed UI	70	29.2

In the reliability analyses, the Fleiss Kappa values of the GPFBQ ranged from 0.65 to 0.92, and the average Fleiss Kappa value was calculated to be 0.81. This result indicates that interobserver agreement is at an ‘excellent’ level.

Table 2 presents a summary of the symptoms associated with pelvic floor disorders and their respective levels of bother. When considering moderate to severe levels of bother for any single symptom, SUI exhibited the highest bother scores. A total of 36% (*n* = 86) of women reported experiencing prolapse symptoms, yet only 15% (15 out of 118) reported experiencing moderate to severe bother. Approximately 32% (*n* = 76) of participants reported AI (anal incontinence), and 18% (*n* = 46) noted obstructive defecation. Additionally, 35% (*n* = 86) of participants reported urinary frequency issues.

**Table 2:** Prevalence of pelvic floor disorder symptoms and bother level.

Scale	No symptoms n (%)	Not bothered n (%)	Little bothered n (%)	Somewhat bothered n (%)	Moderately bothered n (%)	Bothered a lot n (%)
SUI	96 (40)	38 (16)	37 (15)	37 (15)	14 (6)	19 (8)
Frequency	112 (47)	39 (16)	35 (14)	24 (10)	11 (5)	16 (6)
Urgency	116 (49)	36 (15)	32 (14)	25 (10)	16 (7)	10 (4)
UUI	123 (51)	34 (14)	35 (14)	20 (9)	11 (4)	14 (6)
Voiding difficulties	150 (63)	46 (19)	19 (8)	10 (4)	5 (2)	7 (3)
Prolapse	151 (63)	44 (19)	17 (7)	10 (4)	7 (3)	8 (3)
Obs defecation	148 (62)	43 (18)	18 (7)	12 (5)	8 (3)	8 (3)
Anal incontinence	148 (61)	46 (19)	22 (9)	11 (5)	36 (15)	7 (3)
Dyspareunia	96 (40)	140 (59)	0 (0)	0 (0)	0 (0)	0 (0)

The results of the stepwise regression analysis indicated that women’s age, number of births and stress were associated with the presence of incontinence (R2: 21.5%, *p* < 0.001, Durbin-Watson: 1.064; F: 13.755; Table 3).

**Table 3:** Associated factors with the perceived PFD levels of women.

Variables	*B*	SE	(*β*)	*t*	*p*	95% confidence interval	
						Lower limit	Upper limit
Constant	61.97	11.98		5.17	<0.001	38.37	85.57
Age	−1.175	0.310	−0.240	−3.78	<0.001	−1.78	−0.564
Number of birth	13.86	3.73	0.231	3.70	<0.001	6.50	21.23
Presence of stress incontinence	1.28	0.25	−0.295	5.05	<0.001	1.78	0.783
Education level	2.82	1.03	0.164	2.72	0.007	0.781	4.86

R^2^: 21.5%, *p* < 0.001, Durbin-Watson: 1.064, F: 13.755.

A statistically significant correlation was identified between the number of births and the type of urinary incontinence (χ^2^ = 16.60, *p* < 0.05). Specifically, while stress urinary incontinence (SUI) was more prevalent in women who had one to two births, the incidence of mixed urinary incontinence (MUI) increased in women who had three or more births (Table 4). Additionally, the odds ratio (OR) analysis revealed that women who had one to two births were 1.6 times more likely to experience SUI compared to those with three or more births (95% CI: 0.81–3.15), though this result was not statistically significant.

**Table 4:** Distribution of variables by pelvic floor disorder symptoms.

Variables	SUI n (%)	UUI n (%)	Mixed UI n (%)	Significance
BMI				
Normal weight	20 (23)	30 (54)	30 (45)	χ^2^ = 15.32, OR: 1.45, %95 CI: 1.15–1.82 *p* < 0.05
Overweight	45 (52)	18 (32)	27 (40)	
Obese	22 (25)	8 (14)	10 (15)	
Number of birth				
None	20 (20)	34 (49)	16 (23)	χ^2^ = 16.60, *p* < 0.05 OR: 1.6, %95 CI: 0.81–3.15 *p* > 0.05
1–2	51 (51)	27 (38)	22 (31)	
3 or more	29 (29)	9 (13)	32 (46)	

χ^2^: Chi-square test; OR, Odds ratio; CI, Confidence Interval; BMI, Body Mass Index.

A significant correlation was identified between body mass index (BMI) categories and urinary incontinence type (χ^2^ = 15.32, *p* < 0.05). SUI was more common in overweight and obese individuals, while a more balanced distribution between types was observed in normal-weight individuals (Table 4). SUI was more common in overweight and obese individuals, with the odds of experiencing SUI being higher in overweight individuals compared to those with normal weight. In contrast, a more balanced distribution between incontinence types was observed in normal-weight individuals (Table 4). However, further analysis is required to explore the magnitude of the effects and potential confounding variables.

As a result of the qualitative analysis of the data obtained from the interviews with women, 4 categories were identified from the interviews: (1) the impact of UI on daily life, (2) emotional and psychological effects, (3) coping strategies, and (4) treatment approach and expectations. These categories included 8 themes and 21 sub-themes (Table 5).

**Table 5:** Categories, themes and sub-themes related to women’s views and experiences on UI (*n*: 24).

Categories	Theme	Sub-theme
Impacts on daily life	Physical challenges (*n*:18)	Movement Restriction
		Sleep Disorders
		Loss of Energy and Fatigue
	Social impacts (*n*:20)	Social Isolation
		Difficulties in Family and Friend Relationships
		Embarrassment and shyness in society
Emotional and psychological effects	Self-perception (*n*:14)	Loss of Self-Confidence
		Feelings of Shame and Guilt
		Body Image Problems
	Mental state (*n*:16)	Anxiety
		Depression
		Stress Management
Coping strategies	Support seeking behaviors (*n*:19)	Support from Family and Friends
		Getting Professional Help
	Individual coping methods (*n*:24)	Fluid Restriction
		Personal Care and Hygiene Practices
Treatment approach and expectations	Overview of treatment options (*n*:17)	Approach to Drug Therapy
		Willingness for Surgical Intervention
	Expectations and satisfaction (*n*:21)	Expectations from Treatment
		Treatment Process Satisfaction
		Quality of Life Expectations after Treatment

The impact on the daily lives of women with urinary incontinence (UI), their emotional and psychological states, their coping strategies and their perspectives on treatment were analyzed. The results were grouped into four main categories: ‘impact on daily life’, ‘emotional and psychological impact’, ‘coping strategies’ and ‘treatment approach and expectations’ (Table 6).

**Table 6:** The effects of urinary incontinence on women’s daily life, emotional states, coping strategies and treatment expectations.

Categories	Themes	Findings	Participant quotes
Impact on daily life	Physical challenges	The women (*n* = 18) reported experiencing mobility restrictions, sleep disturbances, and fatigue due to UI.	‘… Due to UI, I cannot exercise much, I feel very tired during the day because I wake up frequently at night, and I struggle to complete my daily tasks.’(S12)
	Social effects	The majority of women (*n* = 20) reported experiencing social isolation due to UI, stating that they were unable to spend time with family and friends and that feelings of shame and embarrassment in social settings were prevalent.	‘…Because I have UI, I cannot even visit my neighbors. I cannot meet up with my friends. When I do, I want to go home immediately because I’m afraid I might wet myself.’ (S7)
Emotional and psychological effects	Self-perception	The majority of women (*n* = 14) reported that UI reduced their self-confidence, led to prevalent feelings of shame and guilt, and caused issues with body image.	‘…When I wet myself, I feel ashamed of myself. I do not like myself because I cannot control my bladder. I want to lose weight, but I know I will not succeed; I’ve tried many times, but it has not worked.’ (S10)
	Emotional state	Some women (*n* = 16) reported experiencing anxiety due to UI, undergoing treatment for depression, and struggling with stress management.	‘…Whenever I leave the house, I constantly worry that I will wet myself. Even when my friends come over, I cannot cope with the thought of losing control in front of them. This situation led me to depression, and now I’m on medication, but I still cannot handle these feelings.’ (S3)
Coping strategies	Help-seeking behaviors	The majority of women (*n* = 19) reported consulting friends who also have UI, and when they could not cope, they sought help from healthcare facilities.	‘…My mother, aunt, and cousin were using diapers because of their urinary incontinence. I started using them as well, but since I was struggling to manage it while going to work, I decided to see a doctor.’ (S9)
	Individual coping methods	The women (*n* = 24) reported that they paid close attention to their personal hygiene and restricted fluid intake to prevent urinary incontinence.	‘…When I go out, I only drink one glass of water in the morning and avoid eating or drinking anything until I get home. I carry a bag with three pairs of underwear and two pairs of pants.’ (S5)
Treatment approach and expectations	Perspective on treatment options	The majority of women (*n* = 17) emphasized that surgical treatment could be a definitive solution.	‘…They did not give my neighbor any medication, and she did not get better. Then she had surgery, and all her problems were resolved. Could they perform surgery on me too, so I would not have to bother with taking medication?’ (S9)
	Expectations and satisfaction	The majority of women (*n* = 21) stated that they wished to recover quickly, but noted that the treatment process was prolonged. Despite this, they expressed feeling motivated because they believed their quality of life would improve during the process.	‘…I wish I could recover quickly and go out comfortably, but despite all my efforts, I still experience urinary incontinence. Even though it makes me sad, I feel better than before.’ (S19)

## 4 Discussion

This study provides an in-depth examination of both the physical and psychosocial effects of UI. Unlike most studies in the literature, which tend to focus on the physical symptoms of UI, this study comprehensively evaluates the impact of UI on women’s social lives, emotional well-being, and daily activities using both qualitative and quantitative methods. Particularly, the effects of social isolation and disruptions in self-perception, which are less emphasized in the literature, are thoroughly addressed in this study.

The quantitative findings reveal that SUI poses a significant burden on women. Approximately 36% of participants reported experiencing prolapse symptoms, though only 15% expressed moderate to severe discomfort from these symptoms. Similarly, the rates of participants experiencing anal incontinence and obstructive defecation are notable. These findings highlight the complexity of UI and its varying degrees of impact on individuals. Consistent with previous studies, this research confirmed the significant relationship between UI and factors such as age, number of births, and stress (20, 21). In line with other research, the regression analysis in this study found that these factors increase the risk of incontinence and explained 21.5% of the total variance (22, 23). Higher age and lower education levels were found to be associated with increased risk of UI, consistent with previous findings (24, 25). Our study also highlights that women with one to two births have a higher prevalence of SUI, while those with three or more births exhibit a greater incidence of MUI. In line with earlier research, factors such as mobility restrictions, obesity, and chronic conditions remain significant contributors to UI risk (22, 26).

The qualitative findings provide a deeper understanding of the negative impact of UI on women’s quality of life. Women reported experiencing social isolation, difficulty in daily activities, and loss of self-confidence due to UI. These findings emphasize that UI is not merely a physical issue but also a significant social and emotional burden. The decline in self-perception and psychological outcomes such as anxiety further illustrate the broad impact of UI on mental health. Previous studies have similarly supported the destructive effects of UI on individuals’ social lives and psychological well-being (26–28).

Regarding coping strategies and treatment approaches, the majority of participants expressed positive expectations toward surgical treatment. However, despite the long duration of the treatment process, women remained motivated due to the belief that their quality of life would improve. This is an important factor that enhances individuals’ commitment to the treatment process, and the influence of patient expectations on treatment outcomes in UI has been widely discussed in the literature (28, 29).

The results of this study demonstrate the multifaceted impact of UI on women’s lives and underline the necessity for a comprehensive treatment and support approach to address this condition. In particular, it is clear that social support systems and psychological assistance play a critical role in coping with UI (29, 30). Accelerating treatment processes and improving access to treatments have the potential to significantly enhance women’s quality of life (27, 31).

One of the strengths of this study is its use of the convergent parallel design, which enabled the integration of both qualitative and quantitative data for a comprehensive analysis. This method provided a broader perspective on the effects of UI on women and contributed to a deeper understanding of the findings. While the quantitative data helped elucidate the physical and symptomatic aspects of UI, the qualitative data detailed the social and psychological impacts. Furthermore, this study, conducted in the Turkish context, offers valuable contributions to the literature by considering regional and cultural differences in the effects of UI on women.

However, the study has some limitations. The sample size, particularly in the qualitative data, may limit the generalizability of the findings. Additionally, the high proportion of participants diagnosed with SUI may result in less information about other types of UI. Future studies with larger samples and examinations of different types of incontinence could help address these limitations.

## 5 Conclusion

This study highlights the importance of holistic approaches, including psychosocial support, in managing UI. Healthcare professionals must address the emotional needs of women with UI and develop inclusive, multidisciplinary programs. Revisiting health policies and treatment strategies is essential to implement effective support and rehabilitation for these individuals. UI is influenced by social determinants such as age, education, ethnicity, body mass index, and number of births. Our findings show that stress urinary incontinence is more prevalent among women with one to two births, while mixed urinary incontinence is common in those with three or more births. Overweight and obese women also face a higher risk of stress urinary incontinence, underscoring the need for personalized interventions. Public awareness campaigns should aim to reduce stigma and encourage women to seek care. Culturally sensitive messages and community-based support programs, including pelvic floor exercise workshops and counseling, could significantly improve the quality of life for women with UI.

## Data Availability

The original contributions presented in the study are included in the article/supplementary material, further inquiries can be directed to the corresponding author.

## References

[1] Herdman TH Lopes CT. Supplement to NANDA international nursing diagnoses: Definitions and classification 2021–2023. 12th ed. Georg Thieme Verlag. (2023).

[2] Wallace SL Miller LD Mishra K. Pelvic floor physical therapy in the treatment of pelvic floor dysfunction in women. Curr Opin Obstet Gynecol. (2019) 31:485–93. doi: 10.1097/GCO.0000000000000584, PMID: 31609735

[3] Weber-Rajek M Strączyńska A Strojek K Piekorz Z Pilarska B Podhorecka M. Assessment of the effectiveness of pelvic floor muscle training (PFMT) and extracorporeal magnetic innervation (ExMI) in treatment of stress urinary incontinence in women: a randomized controlled trial. Biomed Res Int. (2020) 2020:1–7. doi: 10.1155/2020/1019872, PMID: 32016111 PMC6988664

[4] Derewiecki T Mroczek M Majcher P Chruściel P. Importance of urinary incontinence problem among women over 40 years of age. Hyg Publ Health. (2015) 50:219–25.

[5] Baran E Akbayrak T Özgül S Nakip G Çinar GN Üzelpasacı E. Musculoskeletal and anthropometric factors associated with urinary incontinence in pregnancy. Physiother Theory Pract. (2022) 38:1789–98. doi: 10.1080/09593985.2021.1878568, PMID: 33522357

[6] Özgül S Gürşen C Toprak Çelenay Ş Baran E Üzelpasacı E Nakip G. Contributory effects of individual characteristics on pelvic floor distress in women with pelvic floor dysfunctions. Physiother Theory Pract. (2022) 40:625–36. doi: 10.1080/09593985.2022.2127137, PMID: 36168816

[7] Weintraub AY Glinter H Marcus-Braun N. Narrative review of the epidemiology, diagnosis and pathophysiology of pelvic organ prolapse. Int Braz J Urol. (2019) 46:5–14. doi: 10.1590/S1677-5538.IBJU.2018.0581, PMID: 31851453 PMC6968909

[8] Dumoulin C Pazzoto Cacciari L Mercier J. Keeping the pelvic floor healthy. Climacteric. (2019) 22:257–62. doi: 10.1080/13697137.2018.1552934, PMID: 30653374

[9] Liu Z Zhou C Munoz A Zhang Y Li X. Validation of a Chinese version for the global pelvic floor bother questionnaire. Arch Gynecol Obstet. (2022) 305:1353–7. doi: 10.1007/s00404-021-06370-7, PMID: 35079874

[10] Astepe BS Köleli I. Translation, cultural adaptation, and validation of Australian pelvic floor questionnaire in a Turkish population. Eur J Obstet Gynecol Reprod Biol. (2019) 234:71–4. doi: 10.1016/j.ejogrb.2019.01.004, PMID: 30665079

[11] Molina-Torres G Amiano-López L Córdoba-Peláez MM Ibáñez-Vera AJ Diaz-Mohedo E. Analysis of the structural characteristics and psychometric properties of the pelvic floor bother questionnaire (PFBQ): a systematic review. J Clin Med. (2022) 11:7075. doi: 10.3390/jcm11237075, PMID: 36498649 PMC9735776

[12] Plano Clark VL. Mixed methods research. J Posit Psychol. (2017) 12:305–6. doi: 10.1080/17439760.2016.1262619

[13] Von Elm E Altman D Egger M Pocock S Gøtzsche P Vandenbroucke J. The strengthening the reporting of observational studies in epidemiology (STROBE) statement: guidelines for reporting observational studies. Rev Esp Salud Publica. (2019) 61:344–9. doi: 10.1016/j.jclinepi.2007.11.008, PMID: 18313558

[14] Tong A Sainsbury P Craig J. Consolidated criteria for reporting qualitative research (COREQ): a 32-item checklist for interviews and focus groups. Int J Qual Health Care. (2007) 19:349–57. doi: 10.1093/intqhc/mzm042, PMID: 17872937

[15] Lorello GR Tewari A Sivagurunathan M Potter E Krakowsky Y Du Mont J. The lived experiences of transgender and gender-diverse people in accessing publicly funded penile-inversion vaginoplasty in Canada. CMAJ. (2024) 196:E816–25. doi: 10.1503/cmaj.231250, PMID: 38955411 PMC11230671

[16] Constantinou CS Georgiou M Perdikogianni M. A comparative method for themes saturation (CoMeTS) in qualitative interviews. Qual Res. (2017) 17:571–88. doi: 10.1177/1468794116686650

[17] Todhunter-Brown A Hazelton C Campbell P Elders A Hagen S McClurg D. Conservative interventions for treating urinary incontinence in women: an overview of Cochrane systematic reviews. Cochrane Database Syst Rev. (2022) 9:CD012337. doi: 10.1002/14651858.CD012337.pub2, PMID: 36053030 PMC9437962

[18] Juhl C Thimm MH Glavind K. Impact on urinary incontinence after management of complications related to a retropubic midurethral sling. Int Urogynecol J. (2023) 34:2767–74. doi: 10.1007/s00192-023-05600-7, PMID: 37470797 PMC10682045

[19] Doğan H Özengin N Bakar Y Duran B. Reliability and validity of a Turkish version of the global pelvic floor bother questionnaire. Int Urogynecol J. (2016) 27:1577–81. doi: 10.1007/s00192-016-3014-8, PMID: 27037562

[20] Liu W Qian L. Risk factors for postpartum stress urinary incontinence: a prospective study. BMC Urol. (2024) 24:42. doi: 10.1186/s12894-024-01430-x, PMID: 38365685 PMC10873983

[21] Ganz M Alessandro C Jacobs M Miller D Diah J Winer A. The role of body mass index and waist circumference in gender-specific risk factors for stress urinary incontinence: a cross-sectional study. Cureus. (2023) 15:e38917. doi: 10.7759/cureus.38917, PMID: 37309351 PMC10257796

[22] Campos NC de Carvalho GM de Santos RA de Brito Oliveira RD de Oliveira Sunemi MM Figueiredo EM. Prevalence, bother, and risk factors associated with occurrence of pelvic floor dysfunctions in young women: a cross-sectional survey. The Journal of Women's & Pelvic Health Physical Therapy. (2024) 48:194–201. doi: 10.1097/JWH.0000000000000305

[23] Palmieri S De Bastiani SS Degliuomini R Ruffolo AF Casiraghi A Vergani P. Prevalence and severity of pelvic floor disorders in pregnant and postpartum women. Int J Gynecol Obstet. (2022) 158:346–51. doi: 10.1002/ijgo.14019, PMID: 34778951

[24] Mou T Brown O Dong S Abbasy S Leung V Simon M. Exploratory mixed methods study on care-seeking behaviors of Asian Americans with pelvic floor symptoms. Int Urogynecol J. (2023) 34:2557–64. doi: 10.1007/s00192-023-05574-6, PMID: 37285090 PMC10246519

[25] Sundqvist C Li X Sundquist K Jansåker F. Sociodemographic disparities and parity in relation to urinary incontinence: a Nationwide primary healthcare cohort study (1997–2018). J Clin Med. (2022) 11:496. doi: 10.3390/jcm11030496, PMID: 35159948 PMC8836927

[26] Peinado Molina RA Hernández Martínez A Martínez Vázquez S Martínez Galiano JM. Influence of pelvic floor disorders on quality of life in women. Front Public Health. (2023) 11:1180907. doi: 10.3389/fpubh.2023.1180907, PMID: 37942254 PMC10629477

[27] Burzynski B Gibala P Soltysiak-Gibala Z Jurys T Przymuszala P Rzymski P. How urinary incontinence affects sexual activity in polish women: results from a cross-sectional study. Int J Environ Res Public Health. (2022) 19:13818. doi: 10.3390/ijerph192113818, PMID: 36360700 PMC9657329

[28] Trinchieri M Perletti G Magri V Stamatiou K Montanari E Trinchieri A. Urinary side effects of psychotropic drugs: a systematic review and metanalysis. Neurourol Urodyn. (2021) 40:1333–48. doi: 10.1002/nau.24695, PMID: 34004020

[29] Ferrari L Gala T Igualada-Martinez P Brown HW Weinstein M Hainsworth A. Multidisciplinary team (MDT) approach to pelvic floor disorders. Continence. (2023) 7:100716. doi: 10.1016/j.cont.2023.100716

[30] Monti M Fischetti M Santangelo G Giannini A D'Oria O Carbone F. Update on surgical treatment of female stress urinary incontinence. Minerva Obstetrics and Gynecology. (2020) 73:140–4. doi: 10.23736/S2724-606X.20.04658-4, PMID: 33103408

[31] Cuccu I Golia D’Augè T Firulli I De Angelis E Buzzaccarini G D’Oria O. Update on genitourinary syndrome of menopause: a scoping review of a tailored treatment-based approach. Life. (2024) 14:1504. doi: 10.3390/life14111504, PMID: 39598302 PMC11595908

